# Immunization coverage and risk factors for failure to immunize within the Expanded Programme on Immunization in Kenya after introduction of new *Haemophilus influenzae *type b and hepatitis b virus antigens

**DOI:** 10.1186/1471-2458-6-132

**Published:** 2006-05-17

**Authors:** Moses Ndiritu, Karen D Cowgill, Amina Ismail, Salome Chiphatsi, Tatu Kamau, Gregory Fegan, Daniel R Feikin, Charles RJC Newton, J Anthony G Scott

**Affiliations:** 1 Wellcome Trust/Kenya Medical Research Institute, Centre for Geographic Medicine Research – Coast, Kilifi, Kenya; 2 Epidemic Intelligence Service, Epidemiology Program Office, Division of Applied Public Health Training, Centers for Disease Control and Prevention Atlanta, GA, USA; 3 Kenya Expanded Programme on Immunization (KEPI), Ministry of Health, Nairobi, Kenya; 4 Kilifi District Public Health Service, Ministry of Health, Kilifi District Hospital, Kenya; 5 Infectious Diseases Epidemiology Unit, Department of Infectious and Tropical Diseases, London School of Hygiene & Tropical Medicine, University of London, UK; 6 Respiratory Diseases Branch, Division of Bacterial and Mycotic Diseases, National Center for Infectious Diseases, Centers for Disease Control and Prevention, Atlanta, GA, USA; 7 Institute of Child Health, University of London, London, UK; 8 Nuffield Department of Clinical Medicine, University of Oxford, John Radcliffe Hospital, Headington, Oxford, UK

## Abstract

**Background:**

Kenya introduced a pentavalent vaccine including the DTP, *Haemophilus influenzae *type b and hepatitis b virus antigens in Nov 2001 and strengthened immunization services. We estimated immunization coverage before and after introduction, timeliness of vaccination and risk factors for failure to immunize in Kilifi district, Kenya.

**Methods:**

In Nov 2002 we performed WHO cluster-sample surveys of >200 children scheduled for vaccination before or after introduction of pentavalent vaccine. In Mar 2004 we conducted a simple random sample (SRS) survey of 204 children aged 9–23 months. Coverage was estimated by inverse Kaplan-Meier survival analysis of vaccine-card and mothers' recall data and corroborated by reviewing administrative records from national and provincial vaccine stores. The contribution to timely immunization of distance from clinic, seasonal rainfall, mother's age, and family size was estimated by a proportional hazards model.

**Results:**

Immunization coverage for three DTP and pentavalent doses was 100% before and 91% after pentavalent vaccine introduction, respectively. By SRS survey, coverage was 88% for three pentavalent doses. The median age at first, second and third vaccine dose was 8, 13 and 18 weeks. Vials dispatched to Kilifi District during 2001–2003 would provide three immunizations for 92% of the birth cohort. Immunization rate ratios were reduced with every kilometre of distance from home to vaccine clinic (HR 0.95, CI 0.91–1.00), rainy seasons (HR 0.73, 95% CI 0.61–0.89) and family size, increasing progressively up to 4 children (HR 0.55, 95% CI 0.41–0.73).

**Conclusion:**

Vaccine coverage was high before and after introduction of pentavalent vaccine, but most doses were given late. Coverage is limited by seasonal factors and family size.

## Background

*Haemophilus influenzae *type b (Hib) is a leading cause of meningitis, septicaemia and pneumonia in young children [1]. Among children aged <5 years, the annual incidence of Hib meningitis in the Gambia was 60/100,000 [2] and of invasive Hib disease in Kenya was 66/100,000 (Cowgill et al, submitted) prior to vaccine introduction. In the Gambia Hib-conjugate vaccine had an efficacy of 95% against invasive Hib disease and prevented >20% of episodes of radiologically-confirmed pneumonia [3].

Hepatitis B virus (HBV) causes 0.5–1.2 million deaths globally each year through chronic hepatitis, cirrhosis and hepatocellular carcinoma [4]. The mean prevalence of chronic HBV infection in Africa is 10.4% but reported prevalence varies depending on communities [5-8]. In the Gambia, among 9-year-old children who received the Hepatitis B vaccine in infancy, vaccine efficacy was 83% against infection with HBV and 93% against chronic infection [8].

In 2001 the Global Alliance for Vaccines and Immunization (GAVI) offered financial support to developing countries to introduce new vaccines and strengthen immunization services. With these funds, Hib-conjugate and Hepatitis B vaccines were introduced into the EPI schedule in Kenya combined with diphtheria, pertussis and tetanus (DPT) antigens as a pentavalent vaccine administered at 6, 10 and 14 weeks of age. Timely administration of these new antigens was considered essential, as invasive Hib disease incidence peaks at 4 months (Cowgill et al, submitted) and vaccination beginning at 6 weeks can prevent early horizontal HBV infection [9].

Cash support of over $11 million for immunization services, particularly for new vaccine clinics, expansion of storage capacity, maintenances of the cold chain, health workers training and community mobilization was designed to increase immunization coverage. At the same time, however, the package volume of the pentavalent vaccine was substantially greater than that of DTP alone, creating potential problems for cold-chain storage and delivery mechanisms, which might have decreased immunization coverage. We set out to determine whether immunization coverage had increased or decreased, to describe the timeliness of immunization and to explore which factors were associated with delayed or missed vaccination, after the introduction of the Hib vaccine. One year after Hib vaccine introduction in Kilifi we observed no detectable impact on Hib disease incidence. Several children who had been admitted to Kilifi Hospital with invasive Hib disease had not been fully vaccinated despite being eligible (Cowgill et al, submitted). We therefore conducted the cluster-sample survey to determine whether the delay in vaccine impact was attributable to imperfect coverage.

## Methods

Kilifi District, the second poorest in the country, is located on the Indian Ocean Coast of Kenya [10]. In the 1999 Kenya Government census it had a population of 544,303. A demographic surveillance study (DSS) was established in 2000 in the sub-population of 210,000 living closest to Kilifi District Hospital, with 4–6 monthly enumeration and registration of vital events. This was the target population for our surveys. Ethical approval for the study was granted by the Kenya Medical Research Institute (KEMRI) National Review Ethical Committee. Verbal consent was sought from participants during invitation to take part in the study. Pentavalent vaccine was distributed rapidly in Kilifi District, and all old DPT vaccine stock recalled, during October 2001. Immunizations are recorded on vaccine cards but children who attend for vaccination without their cards are immunized nonetheless.

### Package volume

We measured the dimensions of the DPT package and the two packages of the pentavalent vaccine that replaced it.

### WHO cluster coverage survey

We simultaneously conducted two WHO-EPI cluster coverage surveys among children of different ages during November-December 2002. The first survey sampled children who were old enough to have completed the 3-dose DPT series prior to introduction of pentavalent vaccine (born between 25^th ^June 2000 and 24^th ^June 2001); the second sampled younger children who should have received 3 doses of pentavalent vaccine (born after 19th September 2001 and at least 14 weeks of age at time of survey).

We followed the WHO-EPI Coverage Survey Manual [11], using the DSS to select at random the starting household for each of 30 clusters. Fieldworkers continued sampling adjacent households until 7 appropriately-aged children were identified in each cluster. Vaccine records or parents'/guardians' (usually mothers') histories were transcribed to WHO-designed forms at the household by trained fieldworkers and double-entered onto a WHO-designed computer programme (WinCosas, Vaccines & Biologicals, WHO, Geneva).

### SRS coverage survey

In March-April 2004 we did a second survey by simple random sampling (SRS) of the DSS database. We ranked >9000 resident children aged 9–23 months (born between 1^st ^Jun 2002 and 31^st ^Jul 2003) by applying random numbers. Starting with the highest ranked child we invited children to take part sequentially until 100 of each sex had been recruited. We asked participants to attend appointments at the research unit where their immunization records were transcribed and digitally scanned. If no immunization record was available or the record was incomplete, the mothers were interviewed using a standard questionnaire. Site of immunization was used to identify the antigens from recall data e.g. only pentavalent vaccine is given into the left lateral thigh. To evaluate the sensitivity of mothers' recall we also interviewed two mothers each day whose children had complete immunization records. The very high response by participants ensured minimal sample bias. Card and questionnaire data were double-entered in Epi-Info (Centers for Disease Control and Prevention, Atlanta). We chose a simple random sample because households in Kilifi are dispersed not clustered in villages [12]; because a DSS register was available; and because a SRS of similar size to the cluster surveys would provide more precise estimates of coverage [13].

### Coverage by clinic register and administrative methods

The numbers of pentavalent vaccine doses delivered to Kilifi District between October 2001 and December 2003 were obtained from the Kenya Expanded Programme on Immunization (KEPI) national stores in Nairobi and provincial stores in Mombasa. The monthly returns to KEPI of the number of doses recorded as given at vaccine clinics in Kilifi District were also collated for the years 2002–3.

Pentavalent vaccine is packaged as a two-dose vial and unused vials must be discarded at the end of each day. We assumed that the daily probability of wasting a single dose in a functioning clinic is 0.5. With 30 vaccination clinics working 20 days a month, there are 7200 vaccine-clinic days leading to 3600 wasted doses per year. As 134,800 doses were delivered to the district in 2002–2003, this suggests a minimum wastage of 5.3%. To adjust for losses due to distribution problems we estimated a total wastage of 10%.

### Population denominator and Geographic Information Systems (GIS) analysis

Annual population growth in rural Kenya in 2003 was 3% and the crude birth rate was 38.6/1000 [14]. We estimated the population of Kilifi District to be 594,774 and 612,618 in the years 2002 and 2003, with birth cohorts of 22,958 and 23,647 respectively. After adjustment for a neonatal mortality rate of 45/1000 live births, this yielded vaccine cohorts of 21,925 and 22,583 children. We digitally mapped subjects' residences in the DSS area and the vaccination clinics in the whole of Kilifi District. The shortest distance from each household to the nearest vaccine clinic was determined directly using ArcGIS v9.0 (ESRI, Redlands, CA).

Data was analysed using STATA v8.2 (Stata Corp, College Station TX). Immunization prevalence was determined for the date of the survey. Timeliness of pentavalent vaccine uptake was determined in the SRS survey using an inverse Kaplan-Meier survival function [15]. In unvaccinated children the time to the next eligible dose was censored on the date of survey. Precision estimates for the cluster survey were modified by a design effect of 2.6 calculated from the data [13]. Administrative coverage was the annual number of doses delivered to vaccine clinics (adjusted for 10% wastage), or administered at clinics, divided by the yearly vaccine cohorts.

A Cox proportional hazards model was fit to recurrent vaccination data to determine the contributions of sex, clinic distance, family size, mother's age and rainfall season to immunization rates. Survival periods were synchronized to start 28 days before each dose was due and separate survival times were entered into the data for each of the three doses for each child, tied by child in a shared-frailty model [16]. Variables were excluded if they did not contribute significantly to model fit (i.e., Likelihood Ratio Test p value >0.05). We tested the proportional hazards assumption using Schoenfeld residuals and by examination of Cox predicted and log-log curves. The resultant hazard ratios are here called age-specific immunization rate ratios or, for brevity, immunization ratios.

Kilifi has rainy seasons from April to July and October to November during which the mean precipitation measured in daily rainfall records in Kilifi from 1993–2004 is = 3 mm. In the other six months mean daily precipitation is <3 mm. A survival period was categorised as rainy if it began during a rainy season month. We also constructed, for each immunization event, the mean daily rainfall for 14 days before each dose was due.

## Results

### Package volume

Pentavalent vaccine is presented in bulk as two packages, 18 cm × 15 cm × 4 cm, providing a total of 200 doses; it replaced a single 500-dose package of DTP, 13 cm × 13 cm × 5 cm. The package volume of the new vaccine is 6.4 times greater per dose than the old vaccine.

### WHO cluster coverage survey

The median (range) age of children born before and after pentavalent vaccine introduction was 695 (524–897) and 251 (99–446) days, respectively; children were born between 25^th ^Jun 2000–20^th ^Jun 2001 and 20^th ^September 2001–1^st ^September 2002 in the two samples. Vaccine cards were retained by 153 (75%) of 203 children before and 181 (88%) of 206 children after pentavalent vaccine introduction (p < 0.001). Combining evidence of vaccine cards and mothers' histories, immunization coverage for three doses of vaccine was 100% (95% CI 95–100) and 91% (95% CI 83–96) in the two samples, respectively (Table 1). For analysis of coverage over time we examined immunization coverage only in children with vaccine cards and at the fixed age of 14 weeks (for doses 1 and 2) or 18 weeks (for dose 3). The coverage for the first dose was 84% (128/153) before, and 93% (168/181) after introduction of pentavalent vaccine (p = 0.01); for the third dose it was 48% (74/153) and 60 % (108/181) respectively (p = 0.04). Immunization coverage for the second dose did not differ significantly. In both samples the median age at immunization was greater than the upper limit of the target age range for immunization (Table 1).

### SRS coverage survey

**Figure 1:**
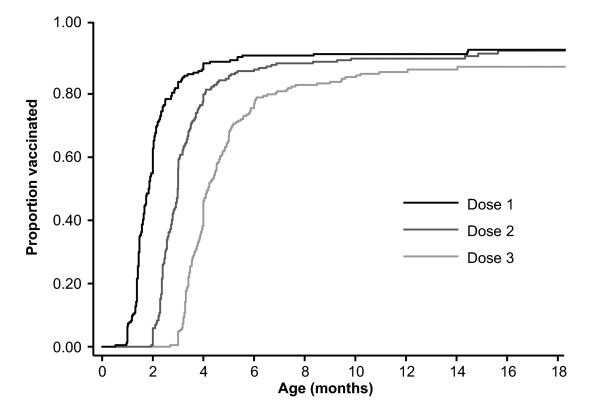
Pentavalent vaccine uptake among 204 in the simple random survey in Kilifi District, Kenya, by inverse Kaplan-Meier survival function

Of two hundred children invited to participate four were late attending the hospital during which interval replacement children were recruited. Of 204 participants, 105 (52%) were male and 165 (81%) had vaccine cards. The median (range) age of mothers and children was 28 years (17–51) and 460 days (277–659), respectively; the children were born between 25^th ^June 2002 and 30^th ^June 2003. Immunization coverage is summarised in Table 2. The sensitivity of immunization histories was estimated in interviews with 18 mothers; all correctly recalled the first dose of vaccine, 17 (94%) recalled the second dose and 16 (88%) recalled the third dose. Timeliness of vaccination is illustrated in the inverse Kaplan-Meier survival curves (Figure 1) and summarised at immunization target dates in Table 3.

### Coverage by clinic register and administrative methods

Clinic registers recorded sufficient immunizations of first, second and third doses of pentavalent vaccine to cover 81%, 78% and 71% of children in Kilifi District, respectively. The numbers of doses of vaccine distributed to Kilifi District from national KEPI stores in 2002 and 2003 were 69,000 and 65,800, respectively; from provincial KEPI records the numbers distributed were 73,300 and 64,600, respectively. Administrative coverage estimates averaged over three doses of vaccine (assuming 10% wastage) were 91% and 93%, by national and provincial records, respectively. For comparison, the mean coverage estimates across all three doses in the SRS survey and clinic register survey were 90% and 76% respectively.

### Factors affecting immunization success

**Figure 2:**
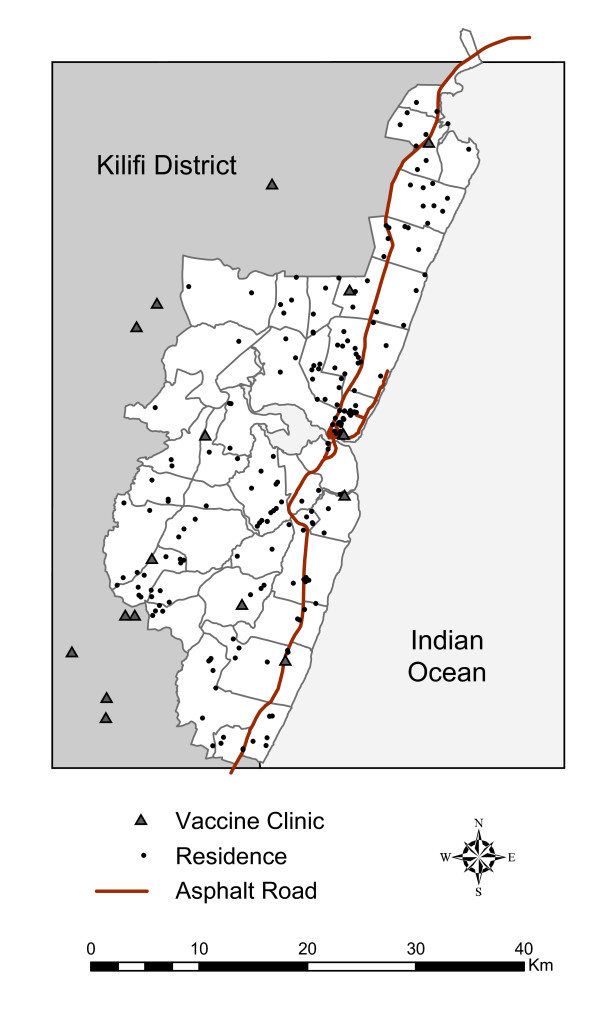
Map of the sub-locations of the Kilifi DSS showing vaccine clinic locations and 204 children's residences in the simple random sample survey

There were 30 government vaccination clinics in the district and 8 within the DSS (Figure 2). The median direct distance to the nearest clinic was 4.19 km (range 0.24–10.1 km). In univariate analyses, season, recent rainfall, distance to clinic, mother's age and family size were each associated with the rate of immunization (Table 4); recent rainfall and mother's age did not contribute to the final multivariable model. The effect of family size showed a gradient which reached a plateau at 4 children. There were no significant interactions between the variables in the final model.

## Discussion

The cluster survey showed that immunization coverage for three doses of DPT/pentavalent vaccine in Kilifi in 2001–2002 was very high (91–100%). The results were underpinned by good retention of vaccine cards in younger children (88%) and reasonable retention in older children (75%). Among older children, only three of 153 children with a vaccine card did not have written evidence confirming receipt of DPT1; among the mothers of these three children, and the 50 children who did not have a vaccine card, every single one reported that her child had received this dose. Results for pentavalent 1 in the younger sample were similar. In both samples an extremely high proportion of mothers reported that their children had received doses two and three.

It is clear from the vaccine card evidence alone that immunization coverage was high but we were concerned about the validity of the mothers recall data which was used to estimate coverage more accurately. The SRS survey attempted to reduce the reliance on mothers' histories and introduced detailed interview about the immunizations. We reasoned that mothers given an appointment at the hospital would have time to find misplaced cards [17] and that interviews conducted at hospital, closely monitored by the investigator, would be superior to field interviews. In the cluster-survey many children were vaccinated long after the target date and some appeared to have been vaccinated too early. In the SRS survey potential transcription errors among outlier dates were counterchecked against a stored digital image of vaccine document. In a previous study from Egypt, data errors were more frequently attributable to interviewer factors and data processing than to maternal recall [17].

Immunization coverage in the SRS survey was also high; 88% of children aged 9–23 months had received three doses of pentavalent vaccine. Despite differences in the methodology, and the fact that the median age of children in the SRS survey was lower, the proportion of children with cards was no greater than in the first survey. As anticipated, however, coverage estimates among those without vaccine cards were slightly lower using hospital-based interviews. The differences between the two surveys, however, may have resulted from changes in coverage over time.

Validation of mothers' histories against complete card entries suggested that mothers in Kilifi have some loss of recall for second and third vaccine doses, indicating that we have slightly underestimated coverage for doses two and three by including recall data. However, in an area with incomplete vaccine card retention coverage is estimated most accurately by combining both card and history data [17]. To analyse only written evidence of immunization from the whole sample would significantly underestimate coverage since vaccine cards are not necessarily retained after the vaccine schedule is completed; to analyse only those who retained a card would overestimate coverage by excluding children who never attend hospital facilities. We included mother's recall data in the analysis of vaccination *timeliness *too because we expected that mothers who did not retain vaccine cards would be less likely to adhere punctually to the vaccine schedule. This turned out to be the case (analysis not shown).

To examine small changes in immunization coverage over time, evidence from mother's memories is invalidated by recall bias. Restricting to card data, and controlling for the potentially confounding effect of age by analysing coverage at 14 or 18 weeks of age we found that coverage was significantly greater for doses 1 and 3 after introduction of Pentavalent vaccine than before. This improvement occurred despite the fact that logistic requirements for Pentavalent vaccine distribution were greater because the per dose package size was more than six times greater than for DTP vaccine alone.

Administrative coverage estimates were slightly higher than those obtained from direct surveys of children, as has been observed elsewhere in Africa [18,19]. The low coverage estimate from clinic registers suggests incomplete reporting at clinic level. Reliance on reported data has more frequently led to overestimation of immunization coverage by developing countries, when compared to survey data [20]. Administrative coverage calculations are particularly sensitive to estimates of vaccine wastage. Assuming that the SRS survey coverage is accurate, and that inaccuracies in the national and provincial administrative estimates are due entirely to vaccine wastage, we can derive indirect estimates of vaccine wastage of 10.9% and 12.9% respectively, slightly over twice the acceptable level for the vaccine.

For a disease like Hib which peaks early in infancy (Cowgill et al, submitted), timeliness of immunization will increase the directly protective effect of immunization. Two doses of Hib-conjugate vaccine are considered sufficient to induce protection against disease [21]. The median age of immunization with two doses in the SRS survey was 3 months, and it was not until after 9 months of age that 90% of children were immunized with a second dose of vaccine. Clearly the protection of a substantial risk group aged <3 months depends entirely on indirect vaccine protection.

In Egypt immunization coverage declined with increasing distance from the vaccination clinics [22]. We observed a similar effect with distance in Kilifi, but the size of this effect was small probably because the median distance to the nearest vaccine clinic was 4 km and therefore vaccination is geographically accessible to the majority of children. Immunization was more strongly associated with annual patterns of rainfall than with the actual precipitation records from the 14 days before each dose was due. This suggests that longer term seasonal changes, such as the requirement to plant crops at a remote small-holding or the increase in costs of public transport fares, which occur at predictable calendar points in relation to anticipated rainfall patterns, are greater impediments to immunization than the rain itself. Parents of large families are less likely to immunize their children. This may simply reflect an association between use of family planning and use of immunization services, or it may indicate that mothers at home are unable to bring their infant for immunization because of the practical difficulties and expense of having other children at home. Kilifi is a poor district in Kenya with high infant mortality rate; better coverage would be expected in other districts and Kenya as a whole.

## Conclusion

In Kilifi District, immunization coverage for two and three doses of the Hib-conjugate and Hepatitis B vaccines at 12 months of age is 91% and 88%, respectively. Coverage estimates derived from national and provincial distribution records corroborate these results. Immunization coverage has not declined over the period of pentavalent vaccine introduction despite a 6-fold increase in the transport and storage requirements. However, at the age of 3 months, only half of all children had received two doses of vaccine. To improve timely coverage it will be necessary to target children from larger families and to explore the impediments to vaccination that occur during seasonal rains.

## Competing interests

The author(s) declare that they have no competing interests.

## Authors' contributions

KDC and JAGS had the idea for the study. MN and KDC collated the data with assistance from SC, AI and TK. The analyses were done by MN, KDC, G F and JAGS. MN wrote the paper with contributions from KDC, DRF, CRJCN and JAGS. All investigators commented on the draft and approved the final version.

## Pre-publication history

The pre-publication history for this paper can be accessed here:



## Figures and Tables

**Table 1:** Immunization coverage by WHO EPI cluster coverage survey in Nov-Dec 2002 in Kilifi District, Kenya

	Before pentavalent (N = 203)*						***After pentavalent (N = 206)^†^***				
	Target age	No. with card evidence	No. with history evidence	Coverage by card or history	Age at immunization (days)^‡^		No. with card evidence	No. with history evidence	Coverage by card or history	Age at immunization (days)^‡^	
vaccine dose	range (days)	of vaccine	of vaccine^§^	(%)	median	range	of vaccine	of vaccine^§^	(%)	median	range
dose 1	39 – 45	150	53	100	54	1–615	176	30	100	53	0–268
dose 2	67 – 73	143	60	100	90	45–657	159	38	96	86	42–268
dose 3	95 – 101	138	65	100	123	78–699	136	51	91	120	70–339

* Children born after 24 June 2000 and before 25 June 2001, aged 18 -30 months, of whom 153 retained vaccine cards

† Children born after 19^th ^September 2001 and at least 14 weeks prior to survey of, aged 3.5 – 12 months, whom 183 retained vaccine cards

‡ Only calculated in children with card evidence

§Includes doses from children with cards who have missing entries for this dose but whose mothers report that they have received the dose nonetheless

**Table 2:** Immunization coverage for selected antigens by simple random sample survey in Mar – Apr 2004 in Kilifi District, Kenya

		***Card evidence in children with cards (N = 165)***		***History evidence in children without cards (N = 39)***		***Card or history evidence combined in all children (N = 204)***	
**Antigen**	number vaccinated	% vaccinated	number vaccinated	% vaccinated	number* vaccinated	% vaccinated	95 % CI
BCG	164	99	31	79	196	96	92 – 98
Birth polio	112	68	29	74	173	85	79 – 89
Polio 1	163	99	29	74	193	95	91 – 97
Polio 2	158	96	26	67	189	93	88 – 96
Polio 3	150	91	23	59	177	87	81 – 91
Penta 1	163	99	29	74	193	95	91 – 97
Penta 2	159	96	27	69	189	93	88 – 96
Penta 3	150	91	24	62	179	88	82 – 92

* Includes children with vaccine cards who have missing entries for vaccines but whose mothers report that they have received the vaccine nonetheless.

**Table 3:** Pentavalent vaccine coverage at different ages among children in Kilifi District estimated from vaccine-card or mother's recall data

**Age of child§**					
Dose of vaccine	6 weeks	10 weeks	14 weeks	18 weeks	12 months
Dose 1	22%	73%	85%	90%	93%
Dose 2	0	15%	63%	81%	91%
Dose 3	0	0	9%	50%	87%

§Children born between 1^st ^Jun 2002 and 31^st ^Jul 2003, aged 9 – 23 months, in SRS coverage survey (N = 204)

**Table 4:** The effect of explanatory factors on age-specific immunization rates with pentavalent vaccine among children in the simple random survey aged 9–23 months in Kilifi District, Kenya

**Variable**	**Univariate immunization rate ratio (95% CI)**		**Adjusted Immunization rate ratio (95 % CI)**	
rainy season*	0.73	(0.61–0.89)	0.73	(0.61–0.89)
recent rainfall >3 mm/day^†‡^	0.94	(0.88–1.00)	-	
distance to vaccine clinic (per 1 km)	0.94	(0.89–0.98)	0.95	(0.91–1.00)
mother's age = 20 years^‡^	0.56	(0.40–0.80)	-	
one child in the family	1		1	
two children in the family	0.71	(0.51–0.97)	0.74	(0.53–1.04)
three children in the family	0.62	(0.45–0.87)	0.61	(0.43–0.87)
four or more children in the family	0.53	(0.41–0.70)	0.55	(0.41–0.73)

* Survival time, the 28 days before the vaccine due date, began during a rainy season month defined as a mean daily rainfall ≥ 3 mm throughout the years 1992–2004.

^†^mean rainfall in the 14 days before the vaccine was due

‡ These variables were excluded from the multivariable model because a Likelihood Ratio Test of their contribution to the model yielded p > 0.05
